# Succinyl-CoA:Mesaconate CoA-Transferase and Mesaconyl-CoA Hydratase, Enzymes of the Methylaspartate Cycle in *Haloarcula hispanica*

**DOI:** 10.3389/fmicb.2017.01683

**Published:** 2017-09-06

**Authors:** Farshad Borjian, Ulrike Johnsen, Peter Schönheit, Ivan A. Berg

**Affiliations:** ^1^Institut für Molekulare Mikrobiologie und Biotechnologie, Westfälische Wilhelms-Universität Münster Münster, Germany; ^2^Mikrobiologie, Fakultät für Biologie, Albert-Ludwigs-Universität Freiburg Freiburg, Germany; ^3^Institut für Allgemeine Mikrobiologie, Christian-Albrechts-Universität zu Kiel Kiel, Germany

**Keywords:** haloarchaea, acetate assimilation, methylaspartate cycle, class III CoA-transferases, enoyl-CoA hydratases

## Abstract

Growth on acetate or other acetyl-CoA-generating substrates as a sole source of carbon requires an anaplerotic pathway for the conversion of acetyl-CoA into cellular building blocks. Haloarchaea (class *Halobacteria*) possess two different anaplerotic pathways, the classical glyoxylate cycle and the novel methylaspartate cycle. The methylaspartate cycle was discovered in *Haloarcula* spp. and operates in ∼40% of sequenced haloarchaea. In this cycle, condensation of one molecule of acetyl-CoA with oxaloacetate gives rise to citrate, which is further converted to 2-oxoglutarate and then to glutamate. The following glutamate rearrangement and deamination lead to mesaconate (methylfumarate) that needs to be activated to mesaconyl-C1-CoA and hydrated to β-methylmalyl-CoA. The cleavage of β-methylmalyl-CoA results in the formation of propionyl-CoA and glyoxylate. The carboxylation of propionyl-CoA and the condensation of glyoxylate with another acetyl-CoA molecule give rise to two C_4_-dicarboxylic acids, thus regenerating the initial acetyl-CoA acceptor and forming malate, its final product. Here we studied two enzymes of the methylaspartate cycle from *Haloarcula hispanica*, succinyl-CoA:mesaconate CoA-transferase (mesaconate CoA-transferase, Hah_1336) and mesaconyl-CoA hydratase (Hah_1340). Their genes were heterologously expressed in *Haloferax volcanii*, and the corresponding enzymes were purified and characterized. Mesaconate CoA-transferase was specific for its physiological substrates, mesaconate and succinyl-CoA, and produced only mesaconyl-C1-CoA and no mesaconyl-C4-CoA. Mesaconyl-CoA hydratase had a 3.5-fold bias for the physiological substrate, mesaconyl-C1-CoA, compared to mesaconyl-C4-CoA, and virtually no activity with other tested enoyl-CoA/3-hydroxyacyl-CoA compounds. Our results further prove the functioning of the methylaspartate cycle in haloarchaea and suggest that mesaconate CoA-transferase and mesaconyl-CoA hydratase can be regarded as characteristic enzymes of this cycle.

## Introduction

The extremely halophilic members of the class *Halobacteria*, known as haloarchaea, are often the predominant habitants in hypersaline environments (Oren, 2002). To keep the cytoplasmic turgor balanced, haloarchaea primarily accumulate molar concentrations of KCl in the cytoplasm. Correspondingly, haloarchaeal enzymes are adapted to these conditions and undergo aggregation or denaturation at low salt concentrations (Lanyi, 1974; Dym et al., 1995). Although haloarchaea are quite uniform in cellular structure and inhabit similar environments, they are surprisingly diverse in their metabolic pathways, particularly regarding the carbon metabolism (Falb et al., 2008; Andrei et al., 2012). Haloarchaeal anaplerotic acetate assimilation pathways are the telling example of this diversity.

**FIGURE 1:**
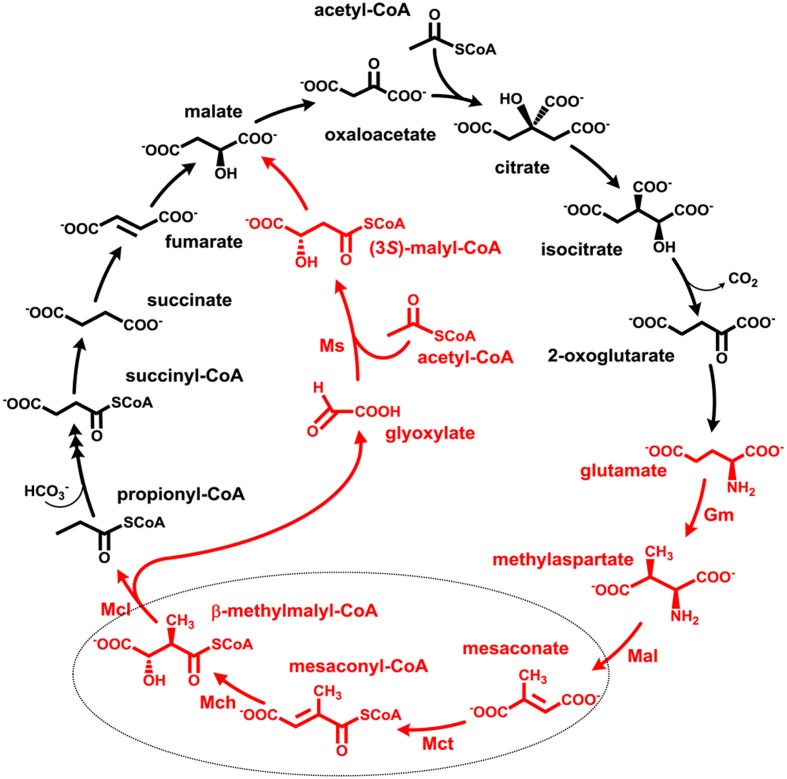
The methylaspartate cycle as studied in *H. hispanica*. The characteristic intermediates of the cycle are shown in red. Gm, glutamate mutase; Mal, methylaspartate ammonia lyase; Mct, succinyl-CoA:mesaconate CoA-transferase; Mch, mesaconyl-CoA hydratase; Mcl, β-methylmalyl-CoA lyase; Ms, apparent malate synthase.

Anaplerotic pathways enable growth on acetate, fatty acids and other compounds metabolized through acetyl-CoA, allowing formation of all cellular building blocks from this central metabolite. Over 92% of all sequenced members of haloarchaea possess genes of anaplerotic acetate assimilation pathways (Borjian et al., 2016). About half of haloarchaea use the glyoxylate cycle discovered in Kornberg and Krebs (1957) that functions in all three domains of life (Serrano et al., 1998). Other haloarchaea use the recently described methylaspartate cycle for acetate assimilation (Khomyakova et al., 2011; Borjian et al., 2016, 2017). In this cycle, one molecule of acetyl-CoA and oxaloacetate are converted to glutamate via the reactions of the tricarboxylic acid (TCA) cycle and glutamate dehydrogenase. The key part of the cycle starts with the rearrangement of glutamate into methylaspartate and its following deamination leading to mesaconate (methylfumarate). Mesaconate is then activated to mesaconyl-CoA, hydrated to β-methylmalyl-CoA and cleaved to propionyl-CoA and glyoxylate. Propionyl-CoA carboxylation leads to methylmalonyl-CoA and subsequently to succinyl-CoA and then (through the TCA cycle) to oxaloacetate, thus closing the cycle. Glyoxylate reacts with another acetyl-CoA molecule yielding malate, the final product of the cycle (**Figure 1**). In sum, the cycle converts two acetyl-CoA molecules into malate. The functioning of the methylaspartate cycle was experimentally shown in *Haloarcula marismortui*, *Haloarcula hispanica* and *Natrialba magadii* and proposed for many other haloarchaea based on the results of bioinformatic analysis of the distribution of its key enzymes (Khomyakova et al., 2011; Borjian et al., 2016, 2017).

Although the methylaspartate cycle functions merely in haloarchaea, its reactions and intermediates can be found in other metabolic pathways functioning in phylogenetically distant microorganisms (Khomyakova et al., 2011). The only reaction that has been found only in the methylaspartate cycle is succinyl-CoA:mesaconate CoA-transferase (further referred to as mesaconate CoA-transferase or Mct) catalyzing mesaconate activation to mesaconyl-C1-CoA. Although Mct has not been characterized so far, the corresponding gene was identified in haloarchaea (Khomyakova et al., 2011; Borjian et al., 2016). The enzyme belongs to class III CoA-transferases (Heider, 2001).

The further conversion of mesaconyl-C1-CoA formed in the Mct reaction is catalyzed by mesaconyl-CoA hydratase (Mch) producing β-methylmalyl-CoA. The enzyme catalyzing this reaction belongs to the family of (*R*)-specific enoyl-CoA hydratases (formerly MaoC family, Teufel et al., 2011). This reaction has previously been identified in two other metabolic pathways: in the autotrophic 3-hydroxypropionate bicycle functioning in *Chloroflexus aurantiacus*, and in the ethylmalonyl-CoA pathway of acetate assimilation present in *Rhodobacter sphaeroides*, *Methylobacterium extorquens* and many other bacteria (Zarzycki et al., 2008). Although *Chloroflexus* and *Rhodobacter* enzymes function in opposite directions under physiological conditions, they share high sequence identity (58 and 73% sequence identity/similarity) (Zarzycki et al., 2008). In contrast, haloarchaeal Mch does not share any significant sequence similarity to *Chloroflexus* and *Rhodobacter* Mch, and its biochemical characterization is of great interest in order to compare catalytic properties of convergently evolved enzymes.

Although the genes for Mct and Mch in haloarchaea are known (*hah_1336* and *hah_1340* in *H. hispanica*, respectively), all our attempts to heterologously produce the corresponding enzymes in *Escherichia coli* resulted in the synthesis of insoluble proteins, and their reactivation was unsuccessful (Khomyakova et al., 2011). In this work, we produced *H. hispanica* enzymes in another haloarchaeon, *Haloferax volcanii*, which lacks the methylaspartate cycle genes and uses the glyoxylate cycle for acetate assimilation. The following purification and characterization of these proteins confirmed their functions and closed the last gaps in characterization of the methylaspartate cycle enzymes.

## Materials and Methods

### Materials

Chemicals were obtained from Fluka (Neu-Ulm, Germany), Sigma–Aldrich (Deisenhofen, Germany), Merck (Darmstadt, Germany), Serva (Heidelberg, Germany), or Roth (Karlsruhe, Germany). Biochemicals were from Roche Diagnostics (Mannheim, Germany), AppliChem (Darmstadt, Germany), or Gerbu (Craiberg, Germany). Materials for cloning were purchased from New England Biolabs (Frankfurt, Germany), Novagen (Schwalbach, Germany), Genaxxon Bioscience GmbH (Biberach, Germany), MWG Biotech AG (Ebersberg, Germany), Biomers (Ulm, Germany), or Qiagen (Hilden, Germany). Primers were synthesized by Sigma–Aldrich (Taufkirchen, Germany). Materials and equipment for protein purification were obtained from GE Healthcare (Freiburg, Germany) or Millipore (Eschborn, Germany).

### Microbial Strains and Culture Conditions

*Haloferax volcanii* strain H1209 was used for the heterologous expression of *H. hispanica* proteins (Allers et al., 2010). This strain was transformed with pTA963 vector carrying *hah_1336* (*mct*) or *hah_1340* (*mch*) under the control of the inducible tryptophan promoter. Expression was performed in complex medium containing the respective compounds according to Allers et al. (2010). *E. coli* strain DH5α (NEB) was grown at 37°C in lysogeny broth (LB) medium (Sambrook et al., 1989). If necessary, ampicillin was added to the cultures to a final concentration of 0.1 mg/ml.

### CoA-Esters

(*R*, *S*)-3-Hydroxy-3-methylglutaryl-CoA and β-methylcrotonyl-CoA were obtained from Sigma–Aldrich. Acetyl-CoA, propionyl-CoA and itaconyl-CoA (mixture of itaconyl-C1-CoA and itaconyl-C4-CoA) were synthesized from their anhydrides and acetoacetyl-CoA from diketene by the method of Simon and Shemin (1953). Itaconyl-C1-CoA and itaconyl-C4-CoA were purified from the mixture of itaconyl-C1-CoA and itaconyl-C4-CoA using high-performance liquid chromatography (HPLC) (Sasikaran et al., 2014). β-Methylmalyl-CoA, (*S*)-malyl-CoA and (*S*)-citramalyl-CoA were synthesized enzymatically with recombinant (*S*)-malyl-CoA/β-methylmalyl-CoA/(*S*)-citramalyl-CoA lyase from *Chloroflexus aurantiacus* (Zarzycki et al., 2009), as described previously (Sasikaran et al., 2014). Crotonyl-CoA, 3-hydroxybutyryl-CoA and a mixture of the mesaconyl-C1-CoA and the mesaconyl-C4-CoA were synthesized chemically from the free acid by the mixed anhydride method of Stadtman (1957). From this mixture, mesaconyl-C1-CoA and mesaconyl-C4-CoA were purified using HPLC (Zarzycki et al., 2009). The dry powders of the CoA-esters were stored at -20°C.

### Preparation of Cell Extracts

Cell extracts were prepared under oxic conditions using an ultrasonic homogenizer Bandelin UW mini20 (BANDELIN Electronic GmbH & Co, Berlin, Germany). Cells (100 mg) were suspended in 0.2 ml of 50 mM Tris(hydroxymethyl)aminomethane/HCl, pH 7.8, including 2 M KCl and 0.1 mg ml^-1^ DNase I in 1.5 ml Eppendorf vials. Cell suspensions were sonicated 3 × 60 s (0.5 s pulse, 70%) on ice, followed by a centrifugation step (14,000 *g*, 4°C, 20 min), and the supernatants (cell extracts) were used for enzyme assays.

### Enzyme Assays

All enzyme assays were performed at 37°C. One unit corresponds to 1 μmol substrate converted per minute.

*Mesaconate CoA-transferase* activity was measured by ultra-performance liquid chromatography (UPLC) as succinyl-CoA- and mesaconate-dependent formation of mesaconyl-CoA. The reaction mixture contained 100 mM Tris/HCl (pH 7.8), 3 M KCl, 5 mM MgCl_2_, 1 mM succinyl-CoA, and cell extract or enzyme. The reaction was started by the addition of 10 mM mesaconate. After appropriate time intervals, 25 μl of the assay mixture was transferred to ice, and the reaction was stopped by addition of 10 μl of 2 M HCl/10% acetonitrile. Protein was removed by centrifugation, and the samples were analyzed by reverse-phase (RP) C_18_ UPLC, as described previously (Sasikaran et al., 2014).

*Mesaconyl-CoA hydratase* was measured in either forward or reverse direction by detecting the formation of β-methylmalyl-CoA from mesaconyl-C1-CoA or mesaconyl-C1-CoA from β-methylmalyl-CoA. For the measurements in the forward direction, the reaction mixture contained 100 mM Tris/HCl (pH 7.8), 3 M KCl, 5 mM MgCl_2_, 1 mM mesaconyl-C1-CoA, and cell extract. For the measurements in the reverse direction, mesaconyl-C1-CoA was replaced by β-methylmalyl-CoA (0.5 mM). The reaction was started by the addition of CoA-ester and stopped, as described above. The products were analyzed by RP-C_18_ UPLC (Sasikaran et al., 2014).

### Determination of *K_m_* and *V_max_* Values for Recombinant Mesaconate CoA-Transferase and Mesaconyl-CoA Hydratase

*K_m_* values for the mesaconate CoA-transferase for succinyl-CoA were measured in the reaction mixture containing 100 mM Tris/HCl (pH 7.8), 3 M KCl, 5 mM MgCl_2_, 10 mM mesaconate, 0.02–5 mM succinyl-CoA, and enzyme. *K_m_* value assays for methylsuccinate and mesaconate where performed, using 1 mM and/or 5 mM succinyl-CoA and 0.1-10 mM of the organic acids. *K_m_* value assays for glutarate and acrylate where performed, using 1 mM succinyl-CoA and 1-100 mM of the organic acids. Assays for 1 mM acetyl-CoA, propionyl-CoA, butyryl-CoA and acetoacetyl-CoA as CoA donor or 10 mM methylmalonate, crotonate, acetoacetate, itaconate, acetate, propionate, butyrate, citrate, citramalate, 2-hydroxyglutarate, citraconate, fumarate, malate and methacrylate as CoA acceptor were performed as described above, using 10 mM mesaconate as CoA acceptor or 1 mM succinyl-CoA as CoA donor, respectively.

*K_m_* values for the mesaconyl-CoA hydratase were measured in the reaction mixture containing 100 mM Tris/HCl (pH 7.8), 3 M KCl, 5 mM MgCl_2_, 0.02-2 mM β-methylmalyl-CoA, 0.05-5 mM mesaconyl-C1-CoA, 0.02-2 mM mesaconyl-C4-CoA, 0.05-5 mM (*S*)-citramalyl-CoA and 0.02-2 mM (*S*)-malyl-CoA. For itaconyl-C1-CoA, itaconyl-C4-CoA, (*R*,*S*)-3-hydroxy-3-methylglutaryl-CoA, β-methylcrotonyl-CoA, crotonyl-CoA and 3-hydroxybutyryl-CoA, 1 mM CoA ester was applied.

### Cloning of *H. hispanica* Genes in *E. coli*

Standard protocols were used for purification, cloning, transformation, and amplification of DNA (Ausubel et al., 1987). Plasmid DNA was isolated with the QIAprep Spin Miniprep Kit (Qiagen).

Primers and restriction enzymes used for the cloning of the *mch* and *mct* genes of *H. hispanica* are listed in Supplementary Table S1. The genes were amplified using Q5 polymerase (NEB). PCR conditions were as follows: 35 cycles of 30-s denaturation at 98°C, 30-s primer annealing and 1 min elongation at 72°C. The PCR products were treated with NdeI and BamHI, and the *mct* and *mch* genes were separately ligated into the expression vector pTA963 containing a sequence encoding an N-terminal His_6_-tag. The plasmids were transformed into *E. coli* DH5α for amplification, followed by their purification and sequencing.

### Heterologous Expression of *mct* and *mch* from *H. hispanica* in *H. volcanii*

The amplified pTA963-*mct* and pTA963-*mch* were used to transform the *H. volcanii* H1209 cells. Growth of transformed cells was performed in Hv-YPC broth (Allers et al., 2010) and the respective cells were used to inoculated 800 ml of Hv-YPC broth. Expression was started (optical density of ∼0.4 at 600 nm) by the addition of 2 mM tryptophan at 42°C. After 18 h of further growth, cells were harvested by centrifugation.

### Purification of Recombinant Enzymes

Frozen cells of the above mentioned expressions were suspended in a double volume of 50 mM Tris/HCl (pH 8.2) containing 2 M KCl, 5 mM imidazole and 0.1 mg ml^-1^ DNase I. The suspensions were passed twice through a chilled French pressure cell at 137 MPa, and the cell lysates were centrifuged for 1 h (100,000 *g*; 4°C). The supernatant was applied at a flow rate of 0.5 ml min^-1^ to a 1-ml Protino Ni-NTA column (Macherey-Nagel) that had been equilibrated with 50 mM Tris/HCl (pH 8.2) containing 2 M KCl and 5 mM imidazole. The column was washed with the same buffer containing 30 mM imidazole at a flow rate of 0.5 ml min^-1^ to elute undesired protein. The recombinant His_6_-tagged enzymes were eluted with the same buffer containing 150 mM imidazole. The enzymes were concentrated using 10K Microsep Advance Centrifugal Device (Pall Corporation, Dreieich, Germany) and stored at 4°C.

Gel filtration chromatography was used for further purification of Mct and Mch and to estimate their native molecular mass. A 24-ml Superdex 200 Increase 10/300 GL column (GE Healthcare) was equilibrated and run with 50 mM Tris/HCl (pH 8.0) containing 2 M KCl with a flow rate of 0.75 ml min^-1^. The column was calibrated with alcohol dehydrogenase (150 kDa), bovine serum albumin (66 kDa), carbonic anhydrase (29 kDa), and cytochrome *c* (12.4 kDa).

### Database Search and Phylogenetic Analysis

Query sequences were obtained from the National Center for Biotechnology Information (NCBI) database. The BLAST searches were performed via NCBI BLASTP server^1^ (Altschul et al., 1990). The amino acid sequences of Mct (*hah_1336*) and Mch (*hah_1340*) from *H. hispanica* were used as queries. The *E*-value cutoff was set at 1e-100 for Mct and 1e-20 for Mch. The amino acid sequences were aligned with sequences from GenBank using CLUSTALW (Thompson et al., 1994) implemented within BioEdit software^2^. The phylogenetic trees were reconstructed using a maximum likelihood algorithm (Felsenstein, 1981) implemented in MEGA6 software (Tamura et al., 2013). 1000 bootstrap replications were conducted to evaluate the reliability of the reconstructed trees. The GenBank accession numbers for the sequences used for the construction of the phylogenetic trees are listed in Supplementary Tables S2, S3.

### Other Methods

Apparent *K*_m_ and *V*_max_ values were calculated using GraphPad Prism4 software. CoA and CoA-esters were identified and quantified by UPLC using an RP-C_18_ column (BEH C_18_, 1.7 μm, 2.1 × 100 mm column, Waters), as described in (Sasikaran et al., 2014). The identification of the CoA-esters was based on co-chromatography with standards and analysis of the UV spectra and retention times (Sasikaran et al., 2014). The UV spectra of mesaconyl-C1-CoA and mesaconyl-C4-CoA are shown in (Zarzycki et al., 2009), of itaconyl-C1-CoA and itaconyl-C4-CoA in Supplementary Figure S1. Protein concentration was determined by two methods. For cell extracts, we used the Bradford method (Bradford, 1976) with bovine serum albumin as a standard. For purified enzymes, protein concentration was determined photometrically (Nanodrop 1000, Peqlab) by measuring an adsorption at 280 nm. Extinction coefficients of the enzymes were calculated from their primary amino acid sequences (Gill and von Hippel, 1989) using the ProtParam online tool^3^. The structural analyses of the enzymes were performed with ProtParam tool in the ExPASy database. DNA sequence determination was performed by GATC Biotech (Constance, Germany). Sodium dodecyl sulfate-polyacrylamide gel electrophoresis (SDS-PAGE) (12.5%) was performed as described by Laemmli (1970). An unstained protein MW marker (Thermo) was used as molecular mass standard (14.4–116 kDa). Proteins were visualized by Coomassie blue staining (Zehr et al., 1989).

Analysis of SDS-gel protein bands was performed by protein mass spectrometry (MS) as following: the MS raw data files were uploaded into the MaxQuant software version 1.4.1.2 (Cox and Mann, 2008), which performs peak detection, generates peak lists of mass error corrected peptides and performs database searches. For the identification of Hah_1336 and Hah_1340, *H. hispanica* and *H. volcanii* database including the corresponding genes from *H. hispanica* was employed, and methionine oxidation and protein amino-terminal acetylation were set as variable modifications. Three miss-cleavages were allowed, enzyme specificity was trypsin/P, and the MS/MS tolerance was set to 0.5 Da. The average mass precision of identified peptides was in general less than 1 ppm after recalibration. Peptide lists were further used by MaxQuant software to identify and relatively quantify proteins using the following parameters: peptide, and protein false discovery rates (FDR) were set to 0.01, maximum peptide posterior error probability (PEP) was set to 0.1, minimum peptide length was set to 6, minimum number peptides for identification and quantitation of proteins was set to two, one of which must be unique, and identified proteins have been requantified.

## Results

### Cloning and Expression of the Genes for Mesaconate CoA-Transferase (*mct*) and Mesaconyl-CoA Hydratase (*mch*)

The 1.19-kb *mct* gene (396 amino acids, predicted molecular mass 43 kDa) and the 1.07-kb *mch* gene (358 amino acids, predicted molecular mass 40 kDa) from *H. hispanica* were cloned to the expression vector pTA-963 containing a tryptophan-inducible promotor p.*tnaA*, resulting in the plasmids pTA-963-mct and pTA-963-mch, respectively. Since these plasmids encode proteins with N-terminal His_6_-tag, their molecular mass is altered by about +1 kDa. Both plasmids were first transformed into *Haloferax volcanii* H1209 for heterologous expression. The heterologously produced enzymes were soluble, as is seen from the results of the detection of Mct/Mch activity in cell extracts of the corresponding *H. volcanii* strains (data not shown).

### Purification of Recombinant Mct and Mch

**FIGURE 2:**
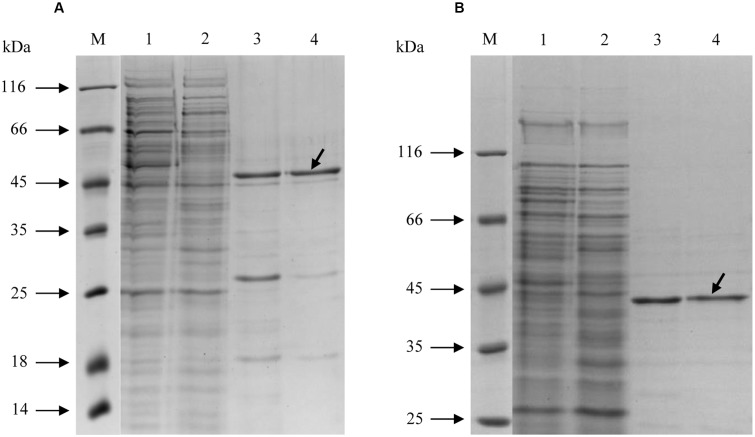
SDS-PAGE (12.5%) of purification steps of recombinant His-tagged **(A)** mesaconate CoA-transferase and **(B)** mesaconyl-CoA hydratase from *H. hispanica*. M, molecular mass markers; lane 1, cell extract without enzyme activity; lane 2, *H. volcanii* cell extract with measurable activity of the heterologously produced enzyme; lane 3, Ni-sepharose column; lane 4, purified enzyme after gel filtration column. The target proteins are shown with arrows.

Both enzymes were purified in a similar manner (**Table 1**). After the 100,000 × *g* centrifugation step, the cell extracts were applied onto a Ni-sepharose affinity column. The active fractions were further purified using gel filtration chromatography, and the concentrated fractions with Mct or Mch activity were used for characterization. Both enzymes could be stored at +4°C for 1 week without loss of activity. However, 3-weeks-incubation resulted in the loss of 20–30% activity. Freezing of the proteins at -20°C led to enzyme inactivation (less than 1% activity remained). The molecular masses of recombinant Mct and Mch in SDS gel (48 and 43 kDa, respectively) were close to the predicted values (44 and 41 kDa, respectively; **Figure 2**). The identity of the purified recombinant proteins was confirmed using gel digestion by trypsin followed by LC-MS/MS (Mct: 28 matched peptides with posterior error probability 0; Mch: 32 matched peptides with posterior error probability 0) (Supplementary Tables S4, S5). Analytical gel filtration indicated a molecular mass of native recombinant enzymes of 71 and 86 kDa for Mct and Mch, respectively, thus suggesting a homodimeric structure for both proteins.

**Table 1:** Purification of recombinant mesaconate CoA-transferase (Mct) and mesaconyl-CoA hydratase (Mch) from *H. hispanica*.

Enzyme	Purification step	Total protein (mg)	Total activity (U)	Specific activity (U mg^-1^)	Enrichment factor	Yield (%)
Mct	Cell extract	66.8	7.05	0.105	1	100
	Nickel column	0.94	6.6	7.02	67	93
	Gel filtration	0.13	2.19	16.9	161	31
Mch	Cell extract	290	107	0.368	1	100
	Nickel column	0.80	96.1	120	327	90
	Gel filtration	0.070	11.01	157	427	10.3

*Activity was tested with 1 mM succinyl-CoA and 10 mM mesaconate (Mct) and with 0.5 mM β-methylmalyl-CoA (Mch)*.

### Catalytic Properties of Recombinant Mct

**FIGURE 3:**
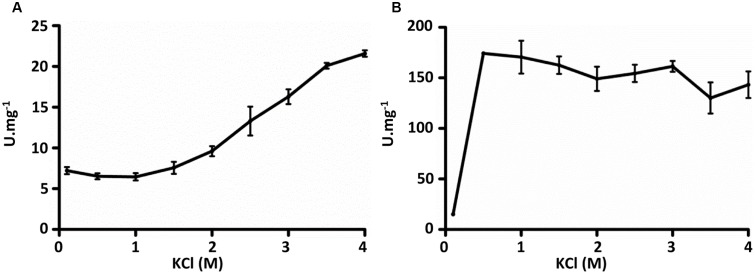
Influence of KCl on activities of recombinant **(A)** mesaconate CoA-transferase and **(B)** mesaconyl-CoA hydratase from *H. hispanica*. Activity was measured with 1 mM succinyl-CoA and 10 mM mesaconate (Mct) and with 0.5 mM β-methylmalyl-CoA (Mch).

The purified recombinant Mct catalyzes the transfer of the CoA moiety from succinyl-CoA (CoA donor) to mesaconate (CoA acceptor) (**Table 2**). Interestingly, only mesaconyl-C1-CoA (and no mesaconyl-C4-CoA) was detected, hence the C1- and C4-carboxyls can efficiently be distinguished in the active center of the enzyme. High activity was detected with methylsuccinate as CoA-acceptor, and only low activity with glutarate, acrylate and itaconate, whereas no activity was found with methylmalonate, crotonate, acetoacetate, acetate, propionate, butyrate, citrate, (*S*)- and (*R*)-citramalate, 2-hydroxyglutarate, citraconate, fumarate, malate and methacrylate. No activity was found with other CoA donors like acetyl-CoA, propionyl-CoA, butyryl-CoA and acetoacetyl-CoA. Mct did not require divalent ions for activity, and addition of EDTA in the reaction mixture did not inhibit the enzyme (**Table 3**). The enzyme was dependent on the presence of KCl and showed highest activity at 4 M KCl (**Figure 3A**).

**Table 2:** Catalytic properties of the recombinant mesaconate CoA-transferase from *H. hispanica*.

Substrate^a^		*V_max_* (U mg^-1^ protein)	*K_m_* (mM)	*k_cat_*/*K_m_* (s^-1^ mM^-1^)
Succinyl-CoA (with 10 mM mesaconate)		69.2 ± 2.6	2.8 ± 0.2	17.8
Methylsuccinate	1 mM succinyl-CoA	11.0 ± 0.6	0.6 ± 0.1	12.4
	5 mM succinyl-CoA	36.8 ± 1.7	2.9 ± 0.3	9.1
Mesaconate	1 mM succinyl-CoA	17.0 ± 0.8	1.3 ± 0.2	9.8
	5 mM succinyl-CoA	45.0 ± 2.1	7.1 ± 0.8	4.6
Glutarate	1 mM succinyl-CoA	18.1 ± 0.8	40.9 ± 3.9	0.32
Acrylate	1 mM succinyl-CoA	6.2 ± 0.3	39.3 ± 4.6	0.12

*The values shown are mean values ± SD of results from typically three independent measurements. ^a^Low activity was found with itaconate (1.65 ± 0.15 U mg^-*1*^ of protein with 1 mM succinyl-CoA and 100 mM itaconate). However, the activity with itaconate did not follow Michaelis–Menten kinetics and increased linearly with increase of itaconate concentration (up to 100 mM). No activity (detection limit ≤ 0.001 U mg^-*1*^) with acetyl-CoA, propionyl-CoA, butyryl-CoA and acetoacetyl-CoA as CoA donor, and methylmalonate, crotonate, acetoacetate, acetate, propionate, butyrate, citrate, (*S*)- and (*R*)-citramalate, 2-hydroxyglutarate, citraconate, fumarate, malate and methacrylate as CoA acceptor*.

**Table 3:** Influence of divalent cations on activity of recombinant Mct and Mch from *H. hispanica*.

Substrate^a^	Mct, %	Mch, %
No addition^b^	100^b^	100^b^
EDTA	109 ± 1	100 ± 5
MgCl_2_	93 ± 1	89 ± 5
MnCl_2_	76 ± 1.5	78 ± 10
NiCl_2_	95 ± 0.5	66 ± 8
CoCl_2_	90 ± 0.2	60 ± 8
CaCl_2_	95 ± 0.1	82 ± 7

*The values shown are mean values ± SD of results from typically three independent measurements. ^a^Activity was tested with 1 mM succinyl-CoA and 10 mM mesaconate (Mct) or 0.5 mM β-methylmalyl-CoA (Mch). The concentration of divalent cations was 5 mM, EDTA 2 mM. ^b^100% activity corresponds to 15.8 ± 0.1 (Mct) and 185 ± 14 (Mch) U mg^-*1*^ of protein*.

### Catalytic Properties of Recombinant Mch

The purified recombinant Mch catalyzed the hydration of mesaconyl-C1-CoA to (2*R*,3*S*)-2-methyl-3-hydroxysuccinyl-C1-CoA (*erythro*-β-methylmalyl-CoA) (**Table 4**); the activity was an order of magnitude higher for the reverse reaction (β-methylmalyl-CoA dehydration). Notably, a similar ratio of the enzyme activity for forward and reverse reactions was observed in *H. hispanica* and *H. marismortui* cell extracts (Khomyakova et al., 2011; Borjian et al., 2016). The enzyme was also active with mesaconyl-C4-CoA, (*S*)-citramalyl-CoA and (*S*)-malyl-CoA (**Table 4**). Nevertheless, enzyme activity was an order of magnitude higher for mesaconyl-C1-CoA/β-methylmalyl-CoA compared to other substrates, thus confirming mesaconyl-C1-CoA and β-methylmalyl-CoA as its physiological substrates/products. No activity was found with itaconyl-C1-CoA, itaconyl-C4-CoA, (*R*, *S*)-3-hydroxy-3-methylglutaryl-CoA and β-methylcrotonyl-CoA. Mch did not require divalent ions for the activity, and addition of EDTA did not inhibit the enzyme (**Table 3**). In contrast to Mct, Mch was most active at 0.5 M KCl (with 82% of activity at 4 M KCl), whereas the activity dropped severely at 0.1 M KCl (**Figure 3B**).

**Table 4:** Catalytic properties of recombinant Mch from *H. hispanica*.

Substrate^a^	*V_max_* (U mg^-1^ protein)	*K_m_* (mM)	*k_cat_*/*K_m_* (s^-1^ mM^-1^)
β-Methylmalyl-CoA	266 ± 13	0.35 ± 0.05	254
Mesaconyl-C1-CoA	20.7 ± 0.7	1.0 ± 0.1	6.9
Mesaconyl-C4-CoA	1.12 ± 0.02	0.18 ± 0.01	2.0
(*S*)-Citramalyl-CoA	1.6 ± 0.1	2.6 ± 0.3	0.2
(*S*)-Malyl-CoA	3.6 ± 0.2	0.32 ± 0.05	3.8

*The values shown are mean values ± SD of results from typically three independent measurements. ^a^Low activity was measured with 1 mM crotonyl-CoA (0.06 U mg^-*1*^) and 1 mM 3-hydroxybutyryl-CoA (0.034 U mg^-*1*^), whereas no activity (detection limit ≤ 0.001 U mg^-*1*^) was detected with itaconyl-C1-CoA, itaconyl-C4-CoA, (*R*, *S*)-3-hydroxy-3-methylglutaryl-CoA, β-methylcrotonyl-CoA*.

### Phylogenetic Analysis of Mct and Mch

**FIGURE 4:**
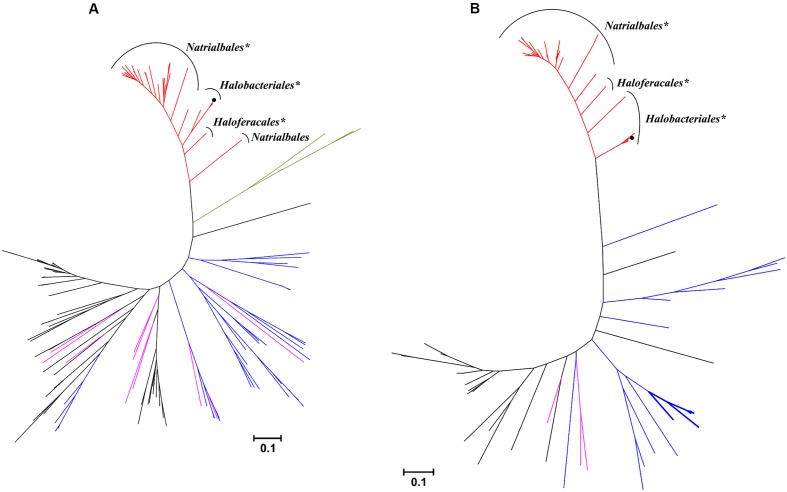
Phylogenetic trees of *H. hispanica* **(A)** mesaconate CoA-transferase and **(B)** mesaconyl-CoA hydratase based on amino acid sequence analysis. Standard NCBI BLASTP searches with mesaconate CoA-transferase (Hah_1336) and mesaconyl-CoA hydratase (Hah_1340) from *H. hispanica* as the queries were performed, and all sequences with *E*-value ≤ 1e-100 for Mct and ≤ 1e-20 for Mch were used for the phylogenetic analysis. Tree topology and evolutionary distances were determined by the maximum-likelihood method with Poisson correction. The scale bar represents a difference of 0.1 substitution per site. The branch colors represent the sequences from haloarchaea, red; α-proteobacteria, purple; β-proteobacteria, black; actinobacteria, blue; *Firmicutes*, green. The groups marked with asterisk (^∗^) possess the complete set of the methylaspartate cycle genes, the bacterial Mch sequences with two domains are shown in bold lines. Please note that bacterial one-domain sequences were aligned with the N-terminal domain of haloarchael Mch.

Among haloarchaea sequenced to date, the *mct* and *mch* homologs were found only in species that possess the methylaspartate cycle (sequence similarity ≥ 69/63% and *E*-value cutoff ≤ 2e-144/1e-135, respectively). No homologs with significant sequence similarity were found in other archaea. In the Mct and Mch phylogenetic trees, the haloarchaeal proteins cluster with bacterial proteins (**Figure 4**). Different archaeal orders (*Natrialbales*, *Haloferacales* and *Halobacteriales*) form separate subclusters in the tree (**Figure 4**). This indicates that *mct* and *mch* were (mainly) transmitted vertically among haloarchaea.

Interestingly, haloarchaeal Mch consists of two (*R*)-specific enoyl-CoA hydratase domains. In the Mch phylogenetic tree, most of homologous proteins to the haloarchaeal enzyme possess only one (*R*)-specific enoyl-CoA hydratase domain homologous to the N-terminal domain of the haloarchaeal protein. Homologous bacterial proteins with two (*R*)-specific enoyl-CoA hydratase domains were found only in high GC actinobacteria *Tsukamurella pseudospumae*, *Rhodococcus* sp. SC4 (2 sequences), *Rhodococcus* sp. LB1, *Rhodococcus opacus* and *Pseudonocardia spinosispora*. In addition, their genomes contain at least one copy of a gene with sequence highly homologous to Mct, although these putative CoA-transferases are not located in vicinity of the Mch homolog. The genes for other key enzymes of the methylaspartate cycle (e.g., methylaspartate ammonia-lyase and glutamate mutase) are missing in their genomes.

## Discussion

In this work, we characterized a novel member of the class III CoA-transferases (Mct) and a member of the (*R*)-enoyl-CoA hydratases family (Mch). In our previous studies (Khomyakova et al., 2011; Borjian et al., 2016), we proposed that the putative haloarchaeal Mct and Mch function in the methylaspartate cycle for mesaconate activation and subsequent hydration of the produced mesaconyl-C1-CoA to β-methylmalyl-CoA. Our study of the catalytic properties of these enzymes confirmed their preliminary annotation.

The turnover number *k_cat_* of Mct with mesaconate and succinyl-CoA (12.2 s^-1^) is comparable to the turnover numbers of other characterized class III CoA-transferases, e.g., acetyl-CoA:oxalate CoA-transferase YfdE from *E. coli* (22 s^-1^), succinyl-CoA:(*S*)-malate CoA-transferase from *C. aurantiacus* (11.4 s^-1^), and succinyl-CoA:3-sulfopropionate CoA-transferase from *Variovorax paradoxus* (37.7 s^-1^) (Friedmann et al., 2006; Schürmann et al., 2013; Mullins et al., 2013). All these CoA-transferases use dicarboxylic acids as substrates. Whereas Mct is highly specific for succinyl-CoA as a CoA-donor, it can activate other carboxylic acids, i.e., methylsuccinate, itaconate, acrylate, and glutarate (**Table 2**). The activation of these substrates can be considered as a side activity of Mct. Indeed, the *K_m_* values for itaconate, glutarate and acrylate were far from the physiological range. Although the catalytic efficiency *k_cat_*/*K_m_* for methylsuccinate was even higher than for mesaconate, this is probably due to the high structural similarity of mesaconate and methylsuccinate. Methylsuccinate is not a physiological substrate of *H. hispanica*, and its participation in any metabolic pathway in haloarchaea is doubtful. Substrate promiscuity was observed for other members of class III CoA-transferases as well. Succinyl-CoA:(*S*)-malate CoA-transferase of *C. aurantiacus* also shows high activity with (*S*)-citramalate (Friedmann et al., 2006), and succinyl-CoA:itaconate CoA-transferase from *Pseudomonas aeruginosa* is highly active with methylsuccinate (Sasikaran et al., 2014). Taken together, the analysis of enzymatic properties of Mct confirms that this protein is a typical member of the class III CoA-transferase superfamily and functions in the methylaspartate cycle for mesaconate activation.

**FIGURE 5:**
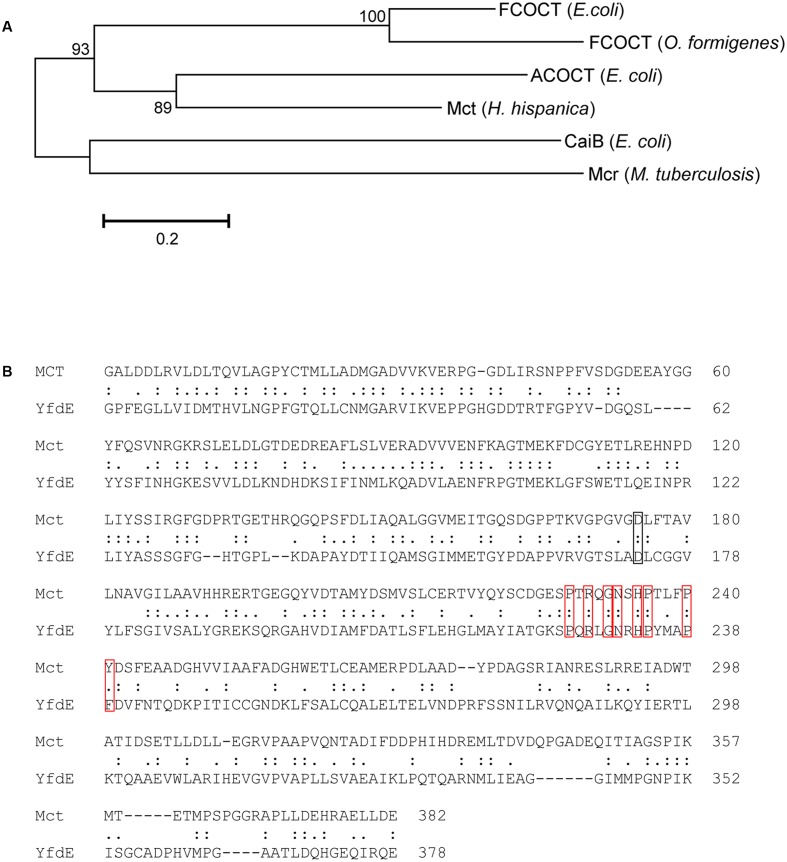
Comparison of *H. hispanica* Mct with other structurally characterized members of class III CoA-transferases. **(A)** Phylogenetic tree of the structurally characterized class III CoA-transferases. The tree is based on amino acid sequence analysis. Tree topology and evolutionary distances were calculated using the maximum-likelihood method with Poisson correction. The scale bar represents a difference of 0.2 substitutions per site. Numbers at nodes indicate the percentage bootstrap values for the clade of this group in 1,000 replications. List of the species and accession numbers are given in Supplementary Table S3. **(B)** Pairwise amino acid sequence comparison of mesaconate CoA-transferase from *H. hispanica* (Mct) and acetyl-CoA:oxalate CoA transferase from *E. coli* (YfdE). The black rectangle indicates the catalytically important Asp169 residue and the red ones indicate the conserved GNxH loop.

The recognition of three distinct classes of CoA-transferases (EC 2.8.3.x) was based on their structure and catalytic mechanisms (Heider, 2001). Both class I and II enzymes form thioester and anhydride intermediates, although their mechanisms are very different. Class I enzymes function via a ping-pong mechanism and mostly use succinyl-CoA or acetyl-CoA as CoA-donor molecules. Class II enzymes catalyze the transfer of an acyl carrier protein with a covalently bound CoA-derivative. Class III enzymes employ a ternary complex mechanism and use CoA-thioesters, being different from both class I and II enzymes. Nevertheless, the known members of class III are highly different in their quaternary structures. Oxalate CoA-transferase, homodimeric acetyl-CoA:oxalate CoA-transferase (YfdE) and butyrobetainyl-CoA:(*R*)-carnitine CoA-transferase (CaiB) are monomeric (Baetz and Allison, 1990; Stenmark et al., 2004; Mullins et al., 2013), while benzoylsuccinate CoA-transferase has an α_2_β_2_ structure (Leutwein and Heider, 2001), phenyllactate CoA-transferase forms a complex with phenyllactyl-CoA hydratase (Dickert et al., 2000), and succinyl-CoA:L-malate CoA-transferase has an (αβ)_6_ structure (Friedmann et al., 2006). Based on size exclusion chromatography data, Mct is a homodimer, like CaiB and YfdE. The amino acid sequence analysis shows 30/45% of identity/similarity to CaiB. Furthermore, most of the residues conserved in CaiB (Stenmark et al., 2004) that were proposed to be important for the folding were also conserved in Mct (Arg16, Gly37, Ala38, Val40, Asp90, Asp169, His185, Thr190, Gly193, but not Leu184; CaiB numbering). Amino acid sequence comparison of Mct with structurally characterized enzymes of class III CoA-transferases shows the highest homology (37/54% of identity/similarity) to YfdE of *E. coli* (**Figure 5A**). Importantly, His233 and its surrounding GNxH loop in the active center of YfdE are also conserved in Mct (**Figure 5B**). Therefore, we suggest that Mct belongs to the ACOCT family of class III CoA-transferases superfamily (Mullins et al., 2013).

Mch is the first characterized archaeal enzyme catalyzing mesaconyl-C1-CoA hydration to *erythro*-β-methylmalyl-CoA. Two characterized bacterial Mchs function in the ethylmalonyl-CoA pathway of acetate assimilation (*R. sphaeroides*) and in the autotrophic 3-hydroxypropionate bi-cycle (*C. aurantiacus*) (Zarzycki et al., 2008). The equilibrium of Mch is on the side of β-methylmalyl-CoA, and the catalytic efficiency *k_cat_/K_m_* for the β-methylmalyl-CoA dehydration reaction is much higher than for mesaconyl-CoA hydration. However, the physiological direction of the reaction catalyzed by Mch in *H. hispanica* is mesaconyl-CoA hydration, similar to the enzyme functioning in the ethylmalonyl-CoA pathway (Zarzycki et al., 2008). Interestingly, the activity of the haloarchaeal enzyme is an order of magnitude lower than the activities of *R. sphaeroides* and *C. aurantiacus* enzymes [*k_cat_* of 89 *s*^-1^ per dimeric enzyme *versus k_cat_* of 1900 *s*^-1^ and 1700 *s*^-1^ for the *R. sphaeroides* and *C. aurantiacus* enzymes, respectively (Zarzycki et al., 2008); **Table 4**]. It appears that the haloarchaeal enzyme is a less efficient catalyst than the bacterial ones. However, a reliable comparison of these enzymes is not possible, as *K_m_* values and substrate specificity of bacterial enzymes are unknown.

Activation of C1 carboxyl of mesaconate in the Mct reaction is a prerequisite for further production of *erythro*-β-methylmalyl-CoA in the Mch reaction. *erythro*-β-Methylmalyl-CoA can further be cleaved into propionyl-CoA and glyoxylate. Mesaconyl-C4-CoA hydration leads to the formation of (*S*)-citramalyl-CoA, which is cleaved into acetyl-CoA and pyruvate (Zarzycki et al., 2009), whereas hydration of non-activated mesaconate results in the formation of (*S*)-citramalate. The last reaction is catalyzed by class I fumarases (Kronen and Berg, 2015; Kronen et al., 2015).

Mch catalyzes also mesaconyl-C4-CoA hydration to (*S*)-citramalyl-CoA and fumaryl-CoA hydration to (*S*)-malyl-CoA (**Table 4**). The catalytic efficiency for (*S*)-malyl-CoA is considerable. Interestingly, this activity could be physiologically relevant, as the *K_m_* value for (*S*)-malyl-CoA is low and *H. hispanica* synthesizes malyl-CoA in its metabolism. Although (to the best of our knowledge) fumaryl-CoA, the product of this reaction, has never being detected as an intermediate in the metabolism, it could further be hydrolyzed to fumarate and CoA, thus forming an alternative route from malyl-CoA to fumarate conversion in the methylaspartate cycle. Although bacterial Mchs have not been tested for malyl-CoA dehydratase activity (Zarzycki et al., 2009), it might be physiologically relevant for the 3-hydroxypropionate bi-cycle. Conversion of succinyl-CoA to fumaryl-CoA (instead of succinate) via a dehydrogenase and subsequent hydration of fumaryl-CoA to (*S*)-malyl-CoA could save 2 enzymatic steps of the 3-hydroxypropionate bi-cycle. Interestingly, we were able to measure considerable (∼5%) (*S*)-malyl-CoA dehydratase activity with purified mesaconyl-CoA hydratase from *C. aurantiacus* (Borjian & Berg, unpublished), and four copies of putative acyl-CoA dehydrogenases were found in the genome of *C. aurantiacus* (Tang et al., 2011).

Although haloarchaeal and bacterial enzymes catalyze the same reaction and belong to the same family of (*R*)-specific enoyl-CoA hydratases, it appears that they evolved convergently from different members of this family. Indeed, they are more closely related to other enoyl-CoA hydratases than to each other (**Figure 4**). While the two mentioned bacterial enzymes share a considerable homology (58/73% of identity/similarity, *E*-value < e-141), haloarchaeal Mch seems to be phylogenetically distinct and shows only 26/41% of identity/similarity with an *E*-value of >5e-13 to the *Rhodobacter* and 24/36% of identity/similarity with an *E*-value of >5e-8 to the *Chloroflexus* enzymes.

As shown in **Figure 3**, Mct and Mch show different behavior toward KCl concentrations. This can be due to the lower theoretical pI of Mct (4.3 for Mct versus 4.7 for Mch), which indicates the presence of higher number of acidic residues on the surface, thus requiring more KCl molecules for the proper protein folding (Mevarech et al., 2000). This is not surprising, as even a slight difference in amino acid sequence can lead to a dramatic shift in halophilicity of a protein. For example, amino acid sequences of nucleoside phosphate kinases of *Haloarcula quadrata* and *Haloarcula sianaiiensis* differ only in one residue but have optimal KCl concentration 1 M and 2 M, respectively (Yamamura et al., 2009).

Taken together, our results show that haloarchaeal Mct and Mch can be regarded as characteristic enzymes of the methylaspartate cycle. They can easily be recognized in a standard BLASTP search, and their presence seems to indicate the presence of a functional methylaspartate cycle in an organism. Indeed, although *Halobacterium salinarum* genome encodes the genes for glutamate mutase and methylaspartate ammonia-lyase, Mct and Mch homologues are absent, and the species is not capable to grow with acetate as a sole carbon source (Falb et al., 2008; Borjian et al., 2016).

## Author Contributions

IB and PS designed the study. IB and FB wrote the manuscript. FB and UJ performed the experimental work.

## Conflict of Interest Statement

The authors declare that the research was conducted in the absence of any commercial or financial relationships that could be construed as a potential conflict of interest.

## Footnotes

1 http://www.ncbi.nlm.nih.gov/BLAST/

2 http://www.mbio.ncsu.edu/BioEdit/bioedit.html

3 http://web.expasy.org/protparam/
