# Supplementary material for: Succinyl-CoA:Mesaconate CoA-Transferase and Mesaconyl-CoA Hydratase, Enzymes of the Methylaspartate Cycle in Haloarcula hispanica
Source: Front Microbiol. 2017 Sep 6;8:1683. doi: 10.3389/fmicb.2017.01683 (PMC5592240; doi:10.3389/fmicb.2017.01683)
Supplement: Supplementary file 1 [file Data_Sheet_1.docx]

***Supplementary material***

**Succinyl-CoA:mesaconate CoA-transferase and mesaconyl-CoA hydratase, enzymes of the methylaspartate cycle
in *Haloarcula hispanica***

**Farshad Borjian,^a,b^ Ulrieke Johnsen,^c^ Peter Schönheit,^c^ Ivan A. Berg^a,b^***

Institut für Molekulare Mikrobiologie und Biotechnologie, Westfälische Wilhelms-Universität Münster, Münster, Germany^a^; Mikrobiologie, Fakultät Biologie, Universität Freiburg, Freiburg, Germany^b^; Institut für Allgemeine Mikrobiologie, Christian-Albrechts-Universität Kiel, Kiel, Germany^c^.

*** Correspondence:** Corresponding Author: ivan.berg@uni-muenster.de

**
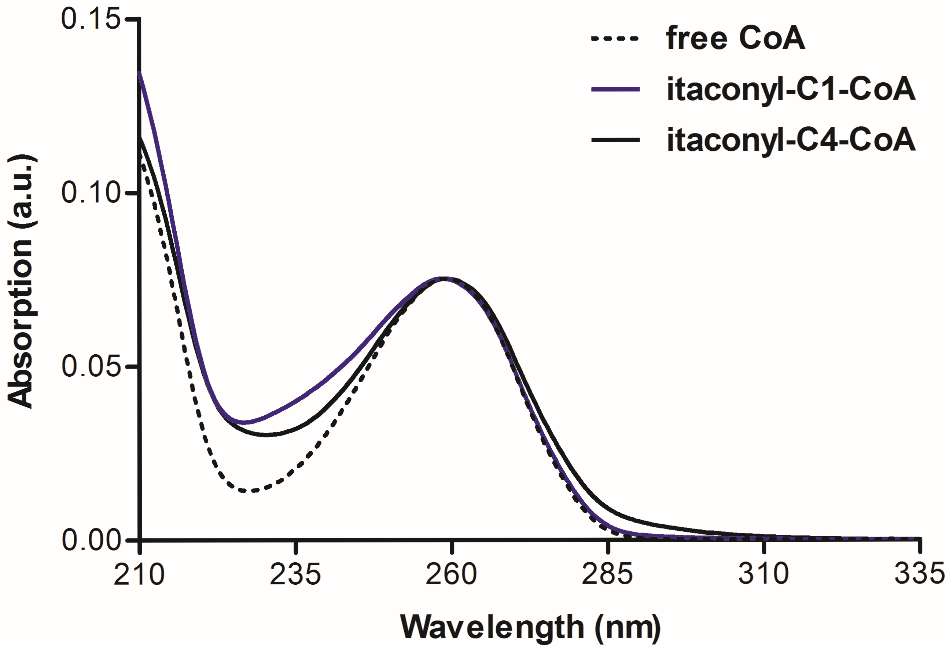
**

**Figure S1. UV spectra of CoA, itaconyl-C1-CoA and itaconyl-C4-CoA.**

**Table S1. List of primers used in this study.** The restriction enzymes used for the cloning are shown in parentheses, the corresponding restriction sites are underlined.

| **Primer** | **Sequence (5’-3’)** | **Used for** |
| --- | --- | --- |
| pTA963-1336F | TATCATATGCACCACCACCACCACCACGGTGCTATGGGTGCGCTTGACGAC (NdeI) | cloning of *hah_1336;mct* |
| pTA963-1336R | ATATAGGATCCGCAGTTGGCGATCAGTCGTC (BamHI) |  |
| pTA963-1340F | TATCATATGCACCACCACCACCACCACGGTGCTATGACTGACTGGACCGATCCC (NdeI) | cloning of *hah_1340;mch* |
| pTA963-1340R | ATATAGGATCCTCATTGGTGTCGCTGAATGTG (BamHI) |  |

**Table S2. Accession numbers for the haloarchaeal sequences used for the construction of the phylogenetic tree shown in the Fig. 4.**

| **Designation in the trees** | **Organism** | **Accession number (Mct)** | **Accession number (Mch)** |
| --- | --- | --- | --- |
| *Halobacteriales** | *Haloarcula hispanica* ATCC 33960 | WP_014040202.1 | WP_014040205.1 |
|  | *Haloarcula amylolytica* JCM 13557 | WP_008309450.1 | WP_008309446.1 |
|  | *Haloarcula argentinensis* DSM 12282 | WP_005534702.1 | WP_005534693.1 |
|  | *Haloarcula californiae* ATCC 33799 | WP_007190472.1 | WP_007190469.1 |
|  | *Haloarcula* sp. CBA1115 | WP_050037966.1 | WP_050037970.1 |
|  | *Haloarcula vallismortis* ATCC 29715 | WP_004516848.1 | WP_004516844.1 |
|  | *Haloarcula marismortui* ATCC 43049 | WP_049938816.1 | WP_049938818.1 |
|  | *Haloarcula sinaiiensis* ATCC 33800 | WP_004961862.1 | WP_004961851.1 |
|  | *Haloarcula japonica* DSM 6131 |  | WP_004592951.1 |
|  | *Halomicrobium katesii* DSM 19301 | WP_018257868.1 | WP_018257865.1 |
|  | *Halomicrobium mukohataei* DSM 12286 | WP_015763926.1 | WP_015763930.1 |
| *Haloferacales** | *Halogranum salarium* B-1 | WP_009374971.1 | WP_009374967.1 |
| *Natrialbales** | *Halopiger salifodinae* | WP_049992038.1 | WP_049992041.1 |
|  | *Natronococcus jeotgali* DSM 18795 | WP_008425952.1 | WP_008425959.1 |
|  | *Natronococcus occultus* SP4 | WP_015320772.1 | WP_015320768.1 |
|  | *Salinarchaeum* sp. Harcht-Bsk1 | WP_020445780.1 | WP_049893856.1 |
|  | *Halopiger xanaduensis* JCM 12890 | WP_013880657.1 | WP_013880661.1 |
|  | *Halobiforma lacisalsi* AJ5 | WP_007142844.1 | WP_007142840.1 |
|  | *Natrialba magadii* ATCC 43099 | WP_004215882.1 | WP_004215878.1 |
|  | *Halostagnicola larsenii* XH-48 | WP_049952491.1 | WP_049952487.1 |
|  | *Natrialba hulunbeirensis* JCM 10989 | WP_006654729.1 | WP_006654733.1 |
|  | *Natronorubrum bangense* JCM 10635 | WP_006064520.1 | WP_006064516.1 |
|  | *Natrinema altunense* JCM 12890 | WP_049899936.1 | WP_007108468.1 |
|  | *Natrialba chahannaoensis* JCM 10990 | WP_006167373.1 | WP_006167381.1 |
|  | *Natronorubrum sulfidifaciens* JCM 14089 | WP_008163863.1 | WP_008163855.1 |
|  | *Natrinema gari* JCM 14663 | WP_008458934.1 | WP_008458924.1 |
|  | *Natrinema pallidum* DSM 3751 | WP_006186757.1 | WP_006186761.1 |
|  | *Haloterrigena salina* JCM 13891 | WP_049914775.1 | WP_008896850.1 |
|  | *Natrialba taiwanensis* DSM 12281 |  | WP_006827678.1 |
|  | *Natrialba aegyptia* DSM 13077 |  | WP_006663952.1 |
|  | *Haloterrigena turkmenica* DSM 5511 | WP_012942778.1 | WP_012942775.1 |
|  | *Haloterrigena limicola* JCM 13563 | WP_049911590.1 | WP_008010317.1 |
|  | *Halobiforma nitratireducens* JCM 10879 | WP_006671564.1 | WP_006671568.1 |
|  | *Natronococcus amylolyticus* DSM 10524 | WP_005555615.1 | WP_049891735.1 |
|  | *Haloterrigena jeotgali* | WP_049964457.1 | WP_049964455.1 |
|  | *Natronorubrum tibetense* GA33 | WP_027119025.1 | WP_006091730.1 |
|  | *Natrinema versiforme* JCM 10478 | WP_006429848.1 | WP_006429852.1 |
|  | *Natrialba asiatica* DSM 12278 | WP_006109415.1 | WP_006109411.1 |
|  | *Natrinema pellirubrum* DSM 15624 | WP_006181787.1 | ELY73982 |
|  | *Haloterrigena thermotolerans* DSM 11522 | WP_006648955.1 | ELZ14880 |
|  | *Natrinema* sp. J7-2 | AFO58513.1 | AFO58509.1 |
|  | *Natronobacterium gregoryi* SP2 | WP_015233825.1 | WP_005576816.1 |
|  | *Natronolimnobius innermongolicus* JCM 12255 | ELY61880.1 | WP_007257584.1 |
|  | *Halovivax ruber* XH-70 | AGB16160.1 | AGB16156.1 |
|  | *Halovivax asiaticus* JCM 14624 | WP_007696440.1 | WP_007696460.1 |
|  | *Halostagnicola larsenii* XH-48 | WP_049955142.1 |  |

**Table S3. Accession numbers for the protein sequences used for the construction of the phylogenetic tree shown in Fig. 5A.**

| **Designation in the tree** | **Related protein** | **Organism** | **Accession** |
| --- | --- | --- | --- |
| ACOCT | Acetyl-CoA:oxalate CoA-transferase | *Escherichia coli* K12 | P76518 |
| CaiB | L-carnitine CoA-transferase | *Escherichia coli* K12 | P31572 |
| FCOCT | Formyl-CoA:oxalate CoA-transferase | *Oxalobacter formigenes* | O06644 |
| FCOCT | Formyl-CoA:oxalate CoA-transferase | *Escherichia coli* K12 | P69902 |
| Mct | Succinyl-CoA:mesaconate CoA-transferase | *Haloarcula hispanica* ATCC 33960 | WP_014040202.1 |
| Mcr | Carnitine dehydratase | *Mycobacterium tuberculosis* ATCC 25618 | O06543 |

**Table S4. List of proteins identified in the band cut from the purified Mct (Fig. 2A).**

| **Protein IDs** | **Organism** | **Peptides^a^** | **PEP^b^** | **Intensity sample** |
| --- | --- | --- | --- | --- |
| Uncharacterized protein | *Haloferax volcanii* ATCC2 9605 | 10 | 7.86E-278 | 0 |
| ArNOG05511 family protein | *Haloferax volcanii* ATCC2 9605 | 18 | 1.84E-256 | 0 |
| Transcriptional regulator | *Haloferax volcanii* ATCC2 9605 | 25 | 6.18E-298 | 798940 |
| **L-carnitine dehydratase/bile acid-inducible protein** | ***Haloarcula hispanica* ATCC 33960** | **28** | **0** | **2.28E+10** |
| Uncharacterized protein | *Haloferax volcanii* ATCC2 9605 | 6 | 7.08E-52 | 0 |
| Htr-like protein | *Haloferax volcanii* ATCC2 9605 | 4 | 4.73E-35 | 0 |
| Deoxyhypusine synthase | *Haloferax volcanii* ATCC2 9605 | 16 | 1.18E-260 | 1E+08 |
| Molybdenum cofactor biosynthesis protein | *Haloferax volcanii* ATCC2 9605 | 11 | 2.55E-155 | 0 |
| CBS domain protein | *Haloferax volcanii* ATCC2 9605 | 17 | 4.90E-248 | 0 |
| Alanine dehydrogenase | *Haloferax volcanii* ATCC2 9605 | 8 | 7.02E-216 | 9061200 |
| Glycerol kinase | *Haloferax volcanii* ATCC2 9605 | 6 | 6.74E-60 | 0 |
| Transcriptional regulator, AsnC family | *Haloferax volcanii* ATCC2 9605 | 3 | 8.76E-07 | 0 |
| Alcohol dehydrogenase | *Haloferax volcanii* ATCC2 9605 | 11 | 1.30E-159 | 1.67E+08 |
| RecJ domain protein | *Haloferax volcanii* ATCC2 9605 | 3 | 2.15E-06 | 0 |
| Ferritin | *Haloferax volcanii* ATCC2 9605 | 5 | 4.14E-38 | 0 |
| ArgK-type transport ATPase | *Haloferax volcanii* ATCC2 9605 | 8 | 5.67E-74 | 0 |
| F420H2:NADP oxidoreductase | *Haloferax volcanii* ATCC2 9605 | 5 | 1.51E-123 | 0 |
| Ribonuclease P protein component 3 | *Haloferax volcanii* ATCC2 9605 | 3 | 1.46E-34 | 0 |
| Indole-3-acetyl-L-aspartic acid hydrolase | *Haloferax volcanii* ATCC2 9605 | 5 | 6.92E-28 | 0 |
| ncharacterized protein | *Haloferax volcanii* ATCC2 9605 | 5 | 1.99E-26 | 0 |
| RecJ domain protein | *Haloferax volcanii* ATCC2 9605 | 5 | 7.46E-22 | 0 |
| Indole-3-acetyl-L-aspartic acid hydrolase | *Haloferax volcanii* ATCC2 9605 | 8 | 1.75E-78 | 14465000 |
| Fructose-bisphosphate aldolase class 2 | *Haloferax volcanii* ATCC2 9605 | 9 | 1.15E-45 | 0 |
| Malate dehydrogenase | *Haloferax volcanii* ATCC2 9605 | 4 | 4.33E-17 | 0 |
| Acetyl-CoA C-acyltransferase | *Haloferax volcanii* ATCC2 9605 | 5 | 5.13E-14 | 15522000 |

^a^Total matched peptides

^b^Error

**Table S5. List of proteins identified in the band cut from the purified Mch (Fig. 2B).**

| **Protein IDs** | **Organism** | **Peptides^a^** | **PEP^b^** | **Intensity sample** |
| --- | --- | --- | --- | --- |
| **MaoC domain protein dehydratase** | ***Haloarcula hispanica* ATCC 33960** | **32** | **0** | **375550** |
| AAA-type ATPase core domain protein | *Haloferax volcanii* ATCC2 9605 | 19 | 2.94E-186 | 7.3E+08 |
| UPF0272 protein HVO_2381 | *Haloferax volcanii* ATCC2 9605 | 10 | 6.73E-59 | 1.39E+08 |
| ABC-type transport system periplasmic substrate-binding protein | *Haloferax volcanii* ATCC2 9605 | 5 | 4.15E-17 | 4682100 |
| Elongation factor 1-alpha | *Haloferax volcanii* ATCC2 9605 | 5 | 2.49E-17 | 1989200 |
| HEAT-PBS family protein | *Haloferax volcanii* ATCC2 9605 | 4 | 9.84E-18 | 1746700 |
| Oxidoreductase (Homolog to thioredoxin-disulfide reductase) | *Haloferax volcanii* ATCC2 9605 | 3 | 2.39E-17 | 0 |
| Deoxyhypusine synthase | *Haloferax volcanii* ATCC2 9605 | 3 | 1.82E-09 | 0 |

^a^Total matched peptides

^b^Error
